# Supported online cognitive behavioural therapy for bulimia nervosa: a study protocol of a randomised controlled trial

**DOI:** 10.1186/s40337-021-00482-w

**Published:** 2021-10-14

**Authors:** Sarah Barakat, Stephen Touyz, Danielle Maloney, Janice Russell, Phillipa Hay, Michelle Cunich, Sharyn Lymer, Marcellinus Kim, Sloane Madden, Jane Miskovic-Wheatley, Sarah Maguire

**Affiliations:** 1 InsideOut Institute, Central Clinical School, The University of Sydney | Sydney Local Health District, Sydney, Australia; 2 School of Psychology, The University of Sydney, Sydney, Australia; 3 Charles Perkins Centre, Faculty of Medicine and Health (Central Clinical School), The University of Sydney, Sydney, Australia; 4 Sydney Local Health District Mental Health Services, Royal Prince Alfred Hospital, Sydney, Australia; 5 Translational Health Research Institute, School of Medicine, Western Sydney University, Sydney, Australia; 6 Camden and Campbelltown Hospitals, South Western Sydney Local Health District, Campbelltown, Australia; 7 Sydney Institute for Women, Children and Their Families, Sydney Local Health District Camperdown, Camperdown, Australia; 8 Sydney Health Economics Collaborative, Sydney Local Health District, Camperdown, Australia; 9 Department of Psychological Medicine, Children’s Hospital at Westmead, Sydney, Australia

**Keywords:** Feeding and eating disorders, Online healthcare, Cost-effectiveness, Cognitive behaviour therapy, Self-help treatment, COVID-19 driven demand for online care

## Abstract

**Background:**

Despite the availability of effective treatments for bulimia nervosa (BN), a number of barriers to accessibility exist. Examples include access to trained clinicians, the expense of treatment, geographical limitations, and personal limitations such as stigma regarding help seeking. Self-help interventions, delivered via a digital platform, have the potential to overcome treatment gaps by providing patients with standardised, evidence-based treatments that are easily accessible, cost-effective, and require minimal clinician support. Equally, it is important to examine the shortcomings of digital interventions when compared to traditional to face-to-face delivery (e.g., high dropout rates) in order to maximise the therapeutic effectiveness of online, self-help interventions.

**Methods:**

A three-arm, multisite randomised controlled trial will be conducted in Australia examining the effectiveness and cost-effectiveness of a newly developed online self-help intervention, *Binge Eating eTherapy (BEeT),* in a sample of patients with full or sub-threshold BN. The BEeT program consists of 10, multimedia sessions delivering the core components of cognitive behaviour therapy. Eligible participants will be randomised to one of three groups: independent completion of BEeT as a purely self-help program, completion of BEeT alongside clinician support (in the form of weekly telemedicine sessions), or waitlist control. Assessments will take place at baseline, weekly, post-intervention, and three-month follow up. The primary outcome is frequency of objective binge episodes. Secondary outcomes include frequency of other core eating disorder behavioural symptoms and beliefs, psychological distress, and quality of life. Statistical analyses will examine treatment effectiveness, feasibility, acceptability and cost effectiveness.

**Discussion:**

There is limited capacity within the mental health workforce in Australia to meet the demand of people seeking treatment for eating disorders. This imbalance has only worsened following outbreak of the COVID-19 pandemic. Further research is required into innovative digital modes of treatment delivery with the capacity to service mental health needs in an accessible and affordable manner. Self-help programs may also appeal to individuals who are more reluctant to engage in traditional face-to-face treatment formats. This study will provide rigorous evidence on how to diversify treatment options for individuals with BN, ensuring more people with the illness can access evidence-based treatment.

The study has been registered with the Australia New Zealand Clinical Trials Registry (ANZCTR Registration Number: ACTRN12619000123145p). Registered 22 January 2019, https://www.australianclinicaltrials.gov.au/anzctr/trial/ACTRN12619000123145.

## Introduction

Bulimia nervosa (BN) is an eating disorder (ED) characterised by recurrent objective binge episodes followed by behaviours intended to compensate for the food eaten during the binge [1]. Binge episodes must involve the consumption of an objectively large amount of food and be accompanied by a sense of lack of control [1]. BN is one of the most common types of EDs globally [2]. According to the Global Burden of Disease Study, the global prevalence rates of BN in 2019 was estimated to be 176.2 per 100,000 people [2]. Epidemiological research conducted on an Australian sample from 1998 to 2008 has identified an upward trend in core symptoms BN including objective binge episodes and extreme dieting [3]. These findings are concerning given the significant physical and psychological impairments imposed by BN [4]. Individuals with BN have been found to report greater functional impairment, higher emotional distress, and lower quality of life than their age-matched peers without the illness [5, 6]. Additionally, significant medical complications are often incurred by individuals with BN as well as economic costs as a result of lower labour force participation, greater absenteeism, and higher health-care costs [2, 7, 8]. The impacts of the illness are only worsened by personal barriers of shame and stigma associated with disordered eating behaviours, which function to discourage help seeking and result in worsening of untreated symptoms [9]. Recent research has identified an average delay of 8.4 years between the onset of BN symptoms and the first instance of help seeking behaviours, with stigma reported as the most impactful barrier [10].

Individual psychological therapy is recognised as the first-line treatment for adults with BN [11], with cognitive behavioural therapy (CBT) having the strongest evidence base of all therapeutic modalities [12–14]. Unfortunately, on average only 23.2% of individuals with an ED access an evidence-based treatment [15]. Limited availability of trained clinicians and poor scalability of therapist-led CBT have been identified as contributors to the poor translation of evidence-based research to real-world populations [16]. The capacity of clinical services to meet the demand of BN patients is often limited by the structure of therapist-led CBT, with most evidence-based treatments stipulating 20 hours of face-to-face therapist contact across a 20-week period [17, 18]. Although there is emerging evidence to suggest ten-session therapist-led CBT may produce  similar clinical outcomes for BN [19], the predicament of limited treatment accessibility remains for those residing in rural and remote areas with very few ED trained clinicians operating outside of metropolitan regions of Australia [20]. Given the demands on public health resources, those who are forced to seek treatment within the private sector are faced with a significant financial expense [21]. There are also concerns regarding the quality of CBT being delivered in the community with studies reporting that as few as 6% of clinicians adhere to evidence-based manuals [22].

Greater attention must be directed towards adapting evidence-based treatments to fit within more time- and cost-efficient models of healthcare delivery, thereby improving accessibility and fidelity within resource constraints. As part of a stepped care model, lower intensity treatments, such as online self-help programs, can be offered to those with less severe clinical presentations in order to prioritise specialist therapeutic skill for patients with greater symptom complexity. Technology-based interventions allow for numerous patients to simultaneously engage with standardised, evidence-based treatments at any time and location that best suits them [16, 23]. The anonymity of digital interventions may assist with the shame and secrecy experienced in relation to bulimic behaviours and the seeking of treatment [24]. Importantly, the recent COVID-19 pandemic has emphasised the need for a technology revolution in the mental health care system [25–30]. Fortunately, digital platforms have allowed mental health services to meet the needs of patients in lieu of in-person contact, highlighting the convenience and flexibility of online treatments and their capacity to overcome the shortcomings of traditional treatment delivery in the current pandemic situation [26, 27, 31, 32].

Several systematic reviews have concluded that digital self-help interventions are effective in reducing eating disorder behaviours [33–40]. There is evidence to also suggest that the treatment outcomes of self-help interventions are significantly enhanced by the addition of low-intensity support from a clinician, such as a fortnightly, check-in email or phone call [12, 39, 41–43]. Supported self-help interventions only demand one-fifth of the therapist contact hours required for a complete CBT course, meaning they can be delivered in settings with low resources and capacity limits [42]. The provision of clinician support as an adjunct to self-help programs has been suggested as a viable solution to one of the most powerful critiques of digital interventions, that being the high rates of non-adherence [37, 38]. Internet-delivered CBT has the highest dropout rates of all ED treatments, ranging up to 47.2% [44]. Given the potential impact of non-adherence upon the therapeutic outcomes for patients, several reviews have highlighted the need for ongoing development and examination of novel digital treatments, with the aim of reducing the discrepancy in adherence between online platforms and more traditional modes of face-to-face treatment delivery [35, 37, 38].

Two large published studies provide evidence for the use of online CBT-based, self-help programs for individuals with BN. As part of a randomised controlled trial (RCT) conducted by Sánchez-Ortiz et al. [45] significant improvements were reported in a sample of 75 patients with full or subthreshold BN following completion of an eight-session online CBT-based program. Abstinence rates were comparable to those of patients receiving a manual-based CBT self-help intervention and face-to-face CBT program [46, 47]. Similarly, Pretorius et al. [48] observed significant reductions in BN symptomology in response to an online CBT program, supplemented with email support from a clinician and peer support via message boards, which were maintained at six-month follow up. An abridged version of BEeT has also been successfully piloted proving its acceptability, feasibility, and effectiveness in reducing core symptoms of BN [49].

Despite the documented effectiveness of online therapies for ED, the availability and uptake of evidence-based digital interventions in the public has been minimal [50]. Very few of the digital interventions examined in preceding clinical trials uphold the technological sophistication required to support user engagement. For example, very few are available as a smartphone application or employ interactive and personalised functions [35]. Further, the vast majority of ED smartphone applications available in the commercial marketplace have not been developed or evaluated by clinical researchers and provide advice that is not informed by evidence-based approaches [51]. Additionally, to our knowledge none of the current online therapies for EDs are available in Australia, with all programs having been developed and evaluated in the USA, UK, and European countries [35].  Consequently, the pilot study of BEeT is the only known evidence which supports the effectiveness of these technologies within the Australian population [49]. Considering the increasing demand for by-distance treatments in the aftermath of COVID-19, and in order to ensure evidence-based treatments are developed in a timely manner, these questions warrant examination. The Binge Eating eTherapy (BEeT) program was developed to address the need for online CBT-based, self-help programs for BN in Australia and to meet the demand for online therapies with up-to-date digital features which can be safely accessed by the patient themselves.

### Study objectives

This randomized controlled trial will compare three study conditions: (1) independent completion of BEeT as a pure self-help program; (2) completion of BEeT alongside 30-min, weekly clinician support; and (3) waitlist control condition (WLC).

The aims of this current trial are to:

1. Investigate the effectiveness of clinician-supported BEeT compared to pure self-help BEeT and WLC in bringing about significant symptom reduction in individuals with BN or subthreshold BN;
2. Examine site-specific outcomes of treatment implementation of clinician-supported BEeT as a low-intensity, eating disorder treatment intervention within the NSW health system; and
3. Determine the cost-effectiveness of WLC as compared to both pure self-help BEeT and clinician-supported BEeT.

## Methods

The present study protocol is reported according to the SPIRIT checklist [52].

### Trial design and setting

**Fig. 1:**
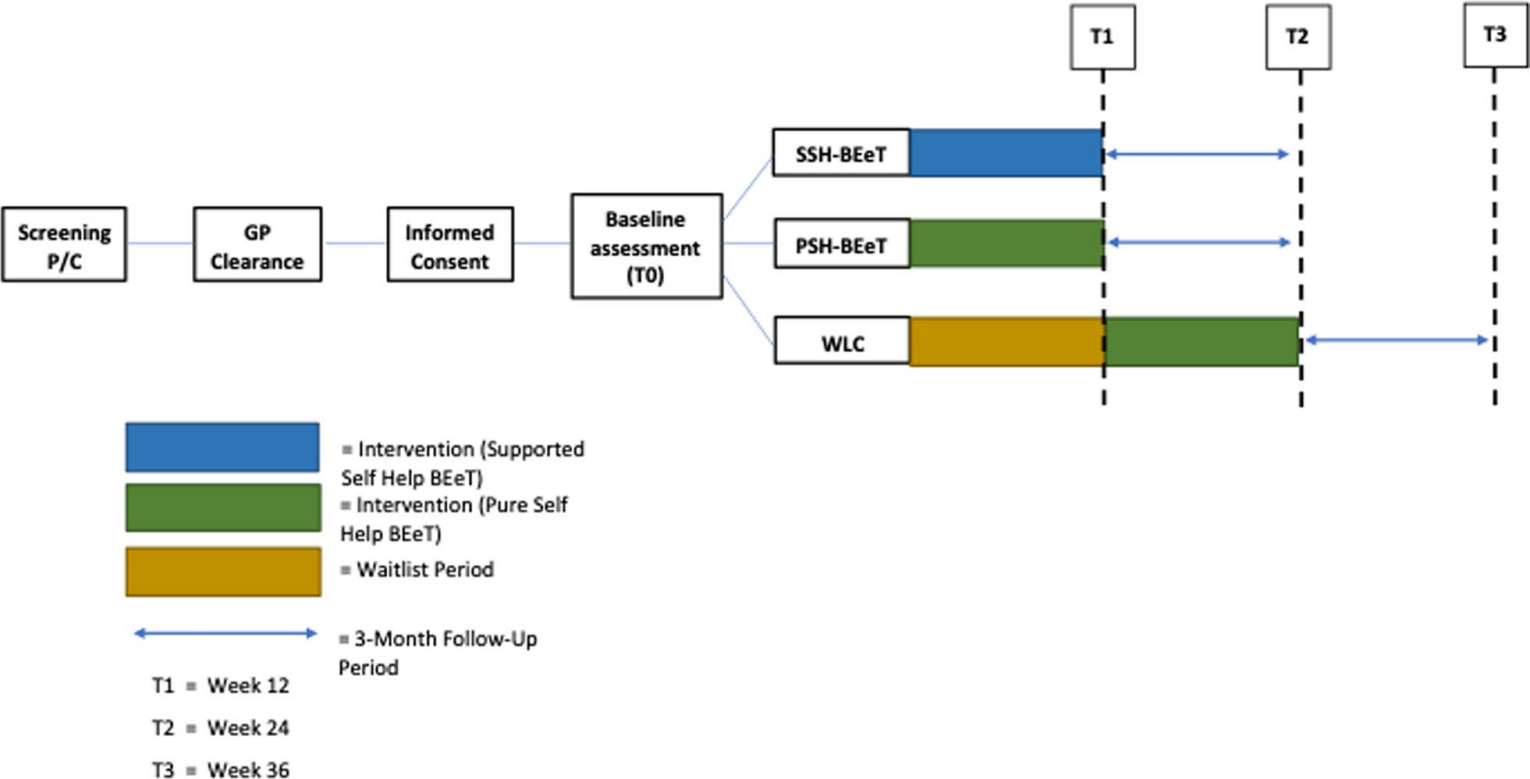
Flow chart of study design

This study is a multisite, three-arm randomized controlled trial inclusive of two active interventions and a WLC. Participants will be recruited from the general community in Australia via advertisements on health websites and social media, referrals made from health professionals, and paper advertisements in mental health and medical clinics. If considered eligible following a screening call, the participant will be instructed to complete a standardised medical assessment with their general practitioner (GP) prior to entry to the study. After providing written informed consent, participants will be randomised to either the WLC condition or one of the intervention conditions, including pure self-help BEeT or clinician-supported BEeT. The intervention is entirely via digital modalities, including (1) *online eTherapy platform* and (2) *secure videoconferencing platform* (only for participants receiving clinician-supported BEeT). Participants randomised to the clinician-supported BEeT condition will engage in telemedicine sessions delivered by clinicians based at one of the following sites: specialist eating disorder outpatient clinic, adult community mental health team, university-based research team, and youth mental health clinic (headspace). All sites are based within New South Wales, Australia. See “Appendix A” for a list of study sites. Participants will be assessed at baseline (T0), weekly, post-intervention (T1: 12 weeks), and at 3-month follow-up (T2). The study commenced in May 2020. The study protocol has been approved by a Human Research Ethics Review Board (HREC #X18-0486). See Fig. 1 for an overview of the study process.

### Eligibility criteria

The primary inclusion and exclusion criteria are listed in Box 1. In addition to these, all participants will be required to complete a standardised medical assessment with their general practitioner (GP) prior to entry in the study. Participants must return clearance form signed by the GP, confirming that they are medically fit to participate, and that the GP agrees to monitor the medical and psychiatric stability of the participant throughout the trial at intervals they deem to be clinically appropriate.

**Box 1:** Eligibility criteria for participation in trial

*Inclusion criteria* Aged 16 years or older; Meets DSM-5 criteria for BN (purging or non-purging type) or Other Specified Feeding or Eating Disorder (OSFED) with bulimic behaviours i.e., must have engaged in objective binge episodes (OBEs) and inappropriate compensatory behaviours (vomiting, excessive laxative or diuretic use, extreme exercise or severe dietary restriction) at least once per week in the preceding 2 months; BMI of 20 or above; Access to a private space with sufficient technology to engage in telemedicine sessions (i.e., digital device with video camera and internet connection) *Exclusion criteria* Currently engaged in ongoing psychological treatment for their eating disorder; Non-proficient English speaker; Serious medical instability; Active suicidality or self-harm behaviours; Severe psychiatric conditions that would interfere with treatment (e.g., psychosis); Pregnant or breast-feeding; Current use of any stimulant medication; Reside outside of Australia	

### Interventions

#### Binge Eating eTherapy (BEeT)

All participants will receive access to BEeT, including both intervention conditions and WLC condition (post 10-week waitlist period). BEeT is an online self-help program that delivers the core components and strategies of CBT proven to be effective in treating eating disorders. BEeT is a 10-week program which consists of 10, one-hour interactive multimedia sessions. BEeT aims to decrease binge eating episodes and compensatory behaviours through the introduction of behavioural techniques which work to establish structured, regular eating patterns as well as cognitive techniques designed to challenge unhelpful thinking patterns related to one’s weight and appearance (See Table 1 for outline of the program components). Users are guided through the online sessions by pre-recorded videos of a therapist. Also included within the sessions are interactive activities to assist users in gaining a personalised understanding of key CBT skills. Each session becomes available every seven days, given that the previous session has been fully completed. Additionally, the program contains an inbuilt digital calendar to promote real-time self-monitoring practice via their digital device using tools such as food diary monitoring, thought challenging, feared foods exposure, and meal planning. The self-monitoring tools uphold several interactive features including a daily or weekly goal setting, scheduled reminders of goals or planned mealtimes, labelling of thoughts and emotions using animated text and images, reminders of coping skills practice, prompting questions to assist with re-framing thoughts, and a visual overview of weekly logs using colour coding to assist with recognising patterns in eating behaviours and thoughts.

**Table 1:** Overview of binge eating etherapy session content

Session	Focus	Key principles
Session 1	Formulation and monitoring eating	Psychoeducation about cognitive-behavioural therapy Introduction to self-monitoring of eating behaviours
Session 2	Eating regularly and planning ahead	Development of personalised case formulation Psychoeducation about the role of restricting food and starvation Regular eating
Session 3	Addressing binges	Key strategies and skills to overcome binge eating and cope with urges to binge The role of triggers in the Binge Eating Cycle
Session 4	Problem solving and motivation	Problem solving Motivational enhancement strategies: pros and cons of change
Session 5	Understanding and noticing thoughts and feelings	Psychoeducation about emotions and unhelpful thinking patterns Emotion regulation skills Introduction to self-monitoring of unhelpful thoughts
Session 6	Coping with thoughts and feelings	Mid-treatment reflection upon strengths and progress Thought challenging
Session 7	Exposure challenges: Feared foods and food rules	Introduction to feared foods and food rules Development of personalised exposure hierarchy How to challenge feared foods and food rules using ‘Exposure Tool’
Session 8	Exposure challenges: Body image	Psychoeducation about body image Identification of exposure tasks to overcome checking and/or avoidance Urge surfing
Session 9	Self-compassion and identifying values and strengths	Strategies to foster greater self-compassion and body acceptance Identifying personal values and strengths external to eating, weight, and shape
Session 10	Review and relapse prevention	Relapse prevention strategies Reflection upon progress and changes in symptoms across treatment Discussion of other treatment options and support

#### Pure self-help BEeT

Participants allocated to the pure self-help BEeT treatment group will be given access to BEeT as described following after their baseline assessment.

#### Clinician-supported BEeT

Participants allocated to the clinician-supported BEeT treatment group will receive access to BEeT in addition to 10, weekly 30-min sessions with a support clinician delivered via a videoconferencing platform. The participant will be instructed to complete each weekly online BEeT session prior to attending the associated telemedicine support session.

The telemedicine sessions adopt the following structure:

1. General check-in regarding participants’ wellbeing (e.g., mood, anxiety, sleep), their eating disorder symptoms (e.g., improved, worsened), and significant events that may have occurred since previous session.
2. Review participants’ understanding of the content introduced in the previous online BEeT session.
3. Review participants’ attempts to complete the learning tasks in their own time, with a particular focus upon debriefing the self-monitoring exercises.
4. Review weekly weigh-in.
5. Preparation for completion of the next online BEeT session.

All clinicians will have completed an online training course in how to deliver a supported self-help CBT intervention for BN, which outlines session by session what needs to be addressed in the support sessions. The clinicians will be existing staff of the service and may have various health qualifications, such as psychology, social work and dietetics. It is expected that participants will meet with their support clinician for a total of approximately five hours across the entire program.

#### Waitlist control

Participants in the WLC will be offered the pure self-help BEeT intervention after a waiting period of ten weeks (T1). During the waiting period they will complete 10, brief weekly psychological assessments (approx. 5 min in length) to monitor eating disorder symptoms, psychological distress and suicidal risk.

### Discontinuation criteria

Participants will be discontinued from the trial if a high level of medical or psychiatric risk is indicated. This includes severe mood instability requiring inpatient hospital admission (e.g., acute suicidality or deliberate self-harm), severe medical complications as confirmed by GP, or if the participant’s BMI drops below 19. Participants will also be discontinued if they are considered to have disengaged from the intervention evidenced by three weeks of non-completion of the online BEeT session or three consecutive missed telemedicine sessions (only for clinician-supported BEeT group).

### Adherence strategies

The BEeT platform contains in-built reminders to prompt engagement with the intervention. These include email notifications to alert participants to when a new online BEeT session is available and an automatic text message at approximately 6 p.m. to participants who have not made a food monitoring entry in the previous 24-h period. In addition, participants will be able to create their own personalised reminders specific to their weekly goals.

Additional efforts to re-engage participants in the pure self-help BEeT or WLC groups will consist of written contact via email to the participant after one week of disengagement, a phone call from researchers or clinicians after two weeks consecutive weeks of disengagement, and a letter to the participant and GP after three consecutive weeks of disengagement. The participant will be given an additional two weeks to reengage before they are discontinued from the trial.

### Contaminant care

Participants will be permitted to seek regular consultation with other health professionals (e.g., dietitian, psychiatrist) whilst actively engaged in the trial, regardless of arm. Concurrent psychological treatment will only be permitted if it is addressing co-occurring mental health difficulties (e.g., anxiety and low mood) and not the eating disorder. Anyone receiving psychological treatment for their ED will be ineligible for the trial.

## Outcomes

Data will be collected via online self-report assessments. Table 2 provides an overview of assessment points and outcome measures.

**Table 2:** Schedule of enrolment, interventions, and assessments according to SPIRIT

	TIMEPOINT**	STUDY PERIOD						
		Enrolment	Allocation	Post-allocation				
		− T_1_	0	T_0_	Weekly monitoring	T_1_	T_2_	T_3_
**Enrolment**	Eligibility screen	X						
	GP Clearance	X						
	Informed consent	X						
	Allocation		X					
**Interventions**	Clinician-supported BEeT							
	Pure self-help BEeT							
	WLC							
**Assessments**	Demographics			X				
	General mental health			X				
	Self-harm and suicidality risk assessment			X	X	X	X	X
	Eating disorder examination questionnaire (EDE-Q)			X		X	X	X
	Eating disorder examination questionnaire-short (EDE-QS)				X			
	Kessler psychological distress scale (K10)			X	X	X	X	X
	Eating disorder quality of life questionnaire (EDQOL)			X		X	X	X
	Three-factor eating questionnaire (TFEQ)			X		X	X	X
	Young schema questionnaire			X		X	X	X
	Body mass index (BMI)			X	X^1^	X	X	X
	European quality of life-5 dimensions-5 levels (ED-5D-5L)			X		X	X	X
	Evaluation of external health services utilised			X		X	X	X
	Implementation surveys					X		
	Working alliance inventory-short revised (WAI-SR)^2^					X		
	Working alliance inventory for online interventions-short form (WAI-TECH-SF)					X		

**T_0_ = baseline assessment, T_1_ = 12 week assessment (post-intervention), T_3_ = 24 week assessment (3 month follow up), T_3_ = 36 week assessment (for WLC only)

^1^BMI measured in fourth week of participation. Also measured weekly from fifth to tenth week for participants where rapid weight loss is indicated

^2^Only administered to clinician-supported BEeT participants

### Primary outcome

The primary outcome is frequency of objective binge episodes as measured by the eating disorder examination questionnaire (EDE-Q) [53]. EDE-Q is a 30-item self-report questionnaire used to assess frequencies of core ED behaviours, including binge episodes and compensatory behaviours, and attitudinal aspects of ED psychopathology across the preceding 28 days. The EDE-Q upholds good reliability (Cronbach’s α = 0.90) [54].

### Secondary outcome measures

#### Measures of eating disorder psychopathology:

*EDE-QS* A shorter 12-item version derived from the EDE-Q, the eating disorder examination questionnaire-short (EDE-QS) assesses ED behaviours and psychopathology severity in the preceding seven days [55]. The EDE-QS is psychometrically and conceptually sound and has good reliability (Cronbach’s α = 0.90) [55, 56].

*EDQOL* The eating disorder quality of life questionnaire (EDQOL) is a disease-specific measure of health-related quality of life [57]. The EDQOL contains 25-items which assess impairment of the ED upon four primary domains: psychological, physical/cognitive, financial, and work/school. The EDQOL has good reliability (Cronbach’s α = 0.90) [57].

*TFEQ* The three factor eating questionnaire (TFEQ) is a 51-item self-report assessment which measures three dimensions of eating behaviour: cognitive restraint of eating, disinhibition/loss of control, and hunger [58]. The TFEQ has been shown to have good reliability across all three dimensions [58].

#### Measures of general mental health psychopathology

*K10* The Kessler Psychological Distress (K10) is a 10-item self-report questionnaire used to monitor the degree of psychological distress [59]. There is evidence to support the reliability of the K10 as a measure of negative emotionality [60, 61, 62].

*YSQ-S3* The young schema questionnaire-short form (YSQ-SF) is a self-report questionnaire which assesses early maladaptive schemas thought to contribute to underlying dysfunctional beliefs and associated psychopathology [63].

*EQ-5D-5L* The five-dimensional European Quality of Life instrument (EQ-5D-5L) will be used to measure the impact of illness upon participants’ mobility, self-care, usual activities, pain/discomfort, and anxiety/depression as well as an overall health score [64]. Utility weights will be obtained and used, along with life years, to calculate Quality Adjusted Life Years (QALYs).

### Other measures regarding the intervention

*WAI-SR* The working alliance inventory-short revised (WAI-SR) is a 12-item self-report assessment which examines the degree of therapeutic alliance experienced by the patient with the clinician across three subscales: bond, task, and goal [65]. It is a widely used measure with good psychometric properties [66].

*WAI-TECH-SF* Working alliance inventory for online interventions-short form (WAI-TECH-SF) is adapted from the WAI-SR and aims to assess the degree of therapeutic alliance between the patient and internet-based self-help programs [67]. It is also a self-report measure with 12-items and has been shown to be a reliable questionnaire [67].

#### Demographics

Demographic characteristics will be collected using a self-designed questionnaire. These include age, gender, occupation, marital status, level of education, income, cultural background/ethnicity, and setting of residence.

#### General mental health

A self-designed questionnaire will collect information regarding the participant’s perception of their current primary and secondary mental health concern (e.g., eating/weight issues, anxiety, depression etc.), history of mental health difficulties, and treatment status (e.g., type of mental health service and treatment being accessed).

#### Self-harm and suicidality risk assessment

Participants’ history of suicidal and self-harming thoughts or behaviours will be assessed using a self-designed questionnaire. It measures severity of suicidality (e.g., suicidal thoughts, suicide attempts etc.) and deliberate self-harm (e.g., urges to self-harm, self-harm actions) in the previous 12 months to 28 days (pre-post treatment and follow up assessments) and previous seven days (weekly assessments).

#### Participant evaluation of external health services utilised

This purpose-built survey will capture information regarding the healthcare resources used (to calculate costs) of participants. Participants will be asked to report the number of health practitioners they have visited in the previous 28-days, including the type/speciality (e.g., GP, psychiatrist, dietitian, physiotherapist etc.), the number of visits/appointments, and the cost per visit.

#### Implementation questionnaire

Self-designed questionnaires will be used to assess treatment implementation from the perspective of participants, clinicians delivering supported BEeT, and trial site managers. The outcome variables include acceptability, adoption, appropriateness, feasibility, fidelity, and sustainability [68]. Feedback will be sought from participants regarding their experience of using the BEeT intervention and clinicians will be asked about their perception of the suitability of the supported BEeT program for patients in their service. Site managers will be asked questions to assess their willingness to embed a supported self-help program, such as BEeT, within their clinical service. Question responses include a combination of 5-point Likert scale and checkbox answers.

#### Compliance

Measures of compliance will be monitored via the online platform on which the BEeT intervention is hosted. These include the number of self-monitoring entries logged per day, the amount of time taken to complete each online BEeT session, and the time between completion of each BEeT session. The compliance data will be included in statistical analyses of intervention response to determine their effects on key clinical and health economic outcomes.

## Participant timeline

See Fig. 1 for the individual participant timeline.

### Sample size

The power calculation is based on EDE-Q outcome data from a recent pilot study of BEeT [49]. The pre- to post-treatment change in objective binge episode frequency, rather than the frequency of purging was chosen as the basis for the power analysis given that not all of the participants in the current study will have engaged in purging behaviours. Accounting for multiple comparisons in the testing procedure, an alpha of 0.05 was adjusted to 0.017 under the Bonferroni method. Using a minimum of 14.9 and maximum of 23.7 for the group means (based on the findings of Barakat et al. [49]), and assuming an adjusted significance level of 0.017, a power of 0.8, and an effect size Cohen’s f = 0.3, a sample size of 48 participants is required in each of the three conditions. By applying a dropout rate of 16% (based upon findings of Barakat et al. [49]), 55 participants are needed in each group. Due to potential resourcing and time limitations, consideration was given to a worst-case scenario of sample size to be recruited. Given this, simulations of the possible impact of reduced sample size was analysed varying the parameters of an effect size (0.25–0.34) and alpha (0.01–0.025). Our worst case scenario indicates that with an effect size of 0.2, alpha of 0.017, and a sample size of 99 participants recruited, the study will have a power of 0.6. This sample size is supported by previous RCTs on eTherapies for eating disorders which have yielded very similar values in their power analyses [69–71]. The power analysis was calculated using STATA (Version 15) [72].

### Randomisation

Randomisation will be performed independently by the National Health and Medical Research Council Clinical Trials Centre, Sydney Australia (NHMRC CTC). The allocation sequence is a permuted block list consisting of blocks randomly varying between size 6 and 9. The study group allocation will be revealed to the researcher via the NHMRC CTC telephone randomisation service. No stratification factors  will be used.

### Blinding

It is not possible to blind participants in this trial given that the difference between the two interventions and the waitlist groups can be detected easily. Blinding of outcome assessors is not applicable given all assessments consist of self-report measures. Data analysts will be blinded to the group allocation of participants.

### Data management and data monitoring

Data collection will be conducted online via two digital platforms: (1) the BEeT intervention platform and (2) RedCAP, a secure electronic data capture platform. All electronic data and confidential study materials, such as participant consent forms, will be stored in an online database via the secure digital platform on which they were collected. Both platforms will only be accessible via a firewall protected website which requires a pre-authorised login and password known only to the Chief Investigator and Associate Investigators involved in data collection/analysis. The BEeT website is hosted on an isolated server, which provides complete privacy and locks the database from the public internet. Data will be de-identified and stored as an SPSS datafile on an enterprise-grade storage platform provided by the University of Sydney. Each participant will be identified in the data set by a unique numerical code. Raw data will remain available on the secure digital platform and output entered routinely into the SPSS datafile as the study progresses.

An audit of files will be conducted regularly to ensure completeness and accuracy of data collection. An independent study monitor is not appointed for this study. The trial monitoring plan is implemented by staff at the University of Sydney. Participant records are selected at random to conduct source data verification on parameters such as demographic data, key outcome variables, informed consent, indicators of risk, and adverse events. Each site will have an on-site monitoring visit at least once per year during the active phase of the study. Members of the steering committee will meet quarterly to discuss any concerns or issues which emerge from the clinical trial monitoring procedures and actions and resolutions are documented.

### Risk management

We anticipate very minimal to no risks to the participant using this intervention. However, it is possible that there may be a slight increase in psychological discomfort during or after completion of the online sessions. To monitor this, participants’ psychiatric risk status will be tracked for the duration of the trial using weekly short psychological assessments. If suicidal thoughts or self-harm is reported, a researcher will contact the participant to complete a risk assessment and provide support as required to ensure their safety. In addition, participant’s weight will be collected at two assessment points (T0 and week four) to monitor medical risks associated with rapid weight loss. If rapid weight loss is indicated, the participant will be asked to provide a weekly measure of their weight for the duration of the trial. Participants will be discontinued from the trial if their BMI is below 19. The GP will also be alerted via email if psychiatric risk rapid or weight loss is identified.

### Statistical methods

Initial exploratory analysis will be conducted to ensure baseline comparability between the two intervention groups and across sites. An intention to treat analysis will be conducted. Multiple imputation analysis will be implemented in accordance with missingness assumptions that fit the data (missing at random vs missing not at random), followed by a sensitivity analysis where appropriate. Generalised linear mixed modelling (GLMM) will be used to compare the effectiveness of pure self-help BEeT versus clinician-supported BEeT vs. WLC, collapsed across settings, using change in objective binge episode frequency as the primary outcome. Co-variates in the model will include baseline information of participant age, illness severity and length of illness. This will allow for assessment of the impact of participant characteristics on the key outcomes. Changes in secondary outcomes, such as psychological distress and quality of life, will also be analysed using a GLMM framework.

Time-to-event statistical methods, in particular survival analysis (cox proportional hazard modelling), will be used to examine non-compliance measures (e.g., time spent using intervention, dropout rates) and the factors impacting it (e.g., symptom severity, comorbid mental health difficulties). GLMM analysis will be used to consider the clinician-supported BEeT subgroup and factors impacting change in the EDE-Q, such as treatment setting and clinician experience to determine if the main outcome is affected by the setting in which treatment is delivered. Descriptive analysis will investigate the embeddedness and implementation of the treatment based on the clinician and the patient questionnaires.

A within-trial economic evaluation will be conducted to determine the cost-effectiveness of clinician-supported BEeT compared to pure self-help BEeT and then to the WLC from a health system perspective using individual-level data. It is crucial that the cost-effectiveness study runs alongside the clinical trial to ensure the health economic evidence is directly related to the outcomes (patient experience, clinical outcomes and quality of life) and costs of study participants (who represent an eligible population in the real world) and can immediately be used by decision-makers in the translation of results into policy and practice (including scaling-up). A cost-utility framework which expresses the main outcomes as Quality Adjusted Life Years (QALYs) will be adopted. The main hypothesis is that the Incremental Cost Effectiveness Ratios (ICERs) are favourable of clinician-supported BEeT at 12 weeks i.e. ICER < $50,000 per QALY gained. We will also assess whether the effect of clinician-supported BEeT is sustained at 3 months and re-calculate the ICERs. A utility-based and disease-generic quality of life measure, EQ-5D-5L, and a disease-specific measure, EDQOL, will be considered in the health economics analysis. Using a health system perspective, we will also be adopted to measure resource use (costs) and other benefits related to the participants and healthcare providers, including implementation costs such as clinician time costs to complete the training course and to deliver the programs. A cost questionnaire (database) will be developed specifically for this clinical trial. This questionnaire will be used to measure the type, amount and value of resources used (and hence adopting a “bottom up” costing approach). A secondary analysis will consider participants’ need for additional technologies or equipment (e.g. laptop, tablet) and time costs for completing treatment sessions. Results will be presented as ICERs which will be calculated as the difference in total costs between clinician-supported compared to pure self-help BEeT (and then compared to WLC) divided by difference in QALYS. Sensitivity analysis will be undertaken.

For the main trial analysis, data will be analysed using IBM SPSS (Version 22.0.0.0, Chicago, IL).

## Discussion

This trial is the first to examine the effectiveness, feasibility, and cost-effectiveness of a CBT-based, online self-help program in an Australian cohort of BN patients in real world healthcare settings. Should the results demonstrate the BEeT program to be an effective intervention, this research has the potential to change the treatment landscape for individuals with BN. Low-intensity programs, such as BEeT, are designed to be integrated within the early stages of a stepped-care model, either as a pure self-help intervention or a clinician-supported intervention [23, 73]. By doing so, there can be improved resourcing in busy clinical services, more BN patients can receive treatment sooner, and hopefully result in a shorter duration of the illness and reduced burden to the individual and healthcare system.

There are several strengths of this trial. Firstly, the three-arm randomised controlled study design allows for assessment of the added benefits of clinician support in comparison to independent engagement with BEeT. Importantly, such outcomes must be considered when defining resource requirements for successful delivery of the intervention, for example the intensity of therapeutic support and necessary level of training of non-specialist clinicians. Secondly, the multisite design provides scope for implementation research through examination of the real-life application of a stepped care approach to EDs within the Australian healthcare system. Data will be collected from the perspective of participants, clinicians, and site managers to determine the site-specific variables affecting implementation of a supported self-help program across multiple outpatient clinics. Implementation science is a rapidly growing field of research, which is vital in facilitating the transfer of evidence-based interventions into clinical practice and community settings [74]. This is particularly important to consider within the domain of digital interventions, with research suggesting poor association between research studies on web-based interventions and meaningful outcomes for patients in clinical settings [75]. Thirdly, our trial includes an online training package for clinicians delivering the intervention, allowing for assessment of its capacity to upskill clinicians to guide patients through a self-help program without previous training in ED treatment.

There are limitations of this trial. To begin, the fidelity of the pure self-help intervention group may be compromised by the degree of contact between the researcher and participant as part of the disengagement protocol. This represents a potential confounding factor as such contact may limit the truly independent nature of the pure self-help condition. For example, follow up phone calls and emails made by researchers may create accountability for the participant to complete the sessions. To account for this in the statistical analyses, the frequency of contact initiated as part of the disengagement protocols will be recorded by research staff. Additionally, this trial started as the COVID-19 outbreak began globally in early to mid-2020. Due to the multisite design of the trial, the COVID-19 pandemic imposed operational challenges and significantly delayed recruitment. Equally, there were advantages of the digital delivery of this intervention in allowing for by-distance mental healthcare within a COVID-safe framework. Also, the mandatory GP medical screening of participants may form a barrier to participation for some individuals and could potentially impinge upon the key advantages of digital treatments, that being it’s accessibility and anonymity. Finally, a high risk of bias may be present due to non-blinding of trial participants [76].

To conclude, this study attempts to address the need for innovative evidence-based digital interventions through the evaluation of a recently developed modern, online platform designed by an interdisciplinary team of digital programmers and designers as well as clinical staff [77]. It is hoped that this study contributes further to current knowledge regarding the ways in which we can effectively adopt an evidence-based mental health strategy which leverages innovative technology.

## Data Availability

Not applicable.

## Appendix A

*List of Study Sites*

- Sydney Local Health District (SLHD)—Peter Beumont Eating Disorders Outpatient Service, Royal Prince Alfred Hospital, Camperdown NSW 2050.
- Northern Sydney Local Health District (NSLHD) Community Mental Health Service, Royal North Shore Hospital, St Leonards NSW 2065.
- Western NSW Local Health District (WNSWLHD) Community Mental Health Service, Orange NSW 2800.
- Charles Perkins Centre-Royal Prince Alfred Clinic (CPC-RPA Clinic) based at the University of Sydney, Camperdown NSW 2050.
- Headspace Liverpool, Liverpool NSW 2170.
- Headspace Wagga Wagga, Wagga Wagga NSW 2650.
- Headspace Camperdown, Camperdown NSW 2050.
- Community Links Wellbeing—ReFrame Program (Wollondilly and Wingecarribee).

## Abbreviations

BN: Bulimia nervosa
ED: Eating disorder
CBT: Cognitive behavioural therapy
BEeT: Binge eating etherapy
WLC: Waitlist control
BMI: Body mass index
GLMM: Generalised linear mixed modelling
QALYs: Quality adjusted life years
ICERs: Incremental cost-effectiveness ratios
